# A Dictionary of Dental Science, Biography, Bibliography and Medical Terminology

**Published:** 1849-07

**Authors:** 


### EDITORIAL DEPARTMENT.

*A Dictionary of Dental Science, Biography, Bibliography and Medical Terminology,* by CHAPIN A. HARRIS, M. D., D. D. S., author of *The Principles and Practice of Dental Surgery, &c., &c.* Philadelphia: Lindsay & Blakison, 1849, pp. 780, royal octavo.

The Dental Profession are under many obligations to Dr. Harris for the valuable Dictionary above referred to. Perhaps no work was ever more needed, and anxiously looked for by the profession for whom it is intended. It has been heretofore as important to the Dental profession as are the Medical and Surgical Dictionaries to their respective sciences; and yet they have been obliged to dispense with that which was next to indispensable while their wants in this particular have been yearly increasing with the advance of their growing science.

This deficiency has long been felt and acknowledged by the profession, and the question has often been asked, who would undertake the task? While they have been thus "deliberating in cool debate," Dr. H. has stepped forward, and with a masterly hand has produced the most ample embodiment of all their cherished wishes.

It not only fulfils the character of a Dictionary, but it partakes largely of that of an *Encyclopædia of Dental Science;* so that with a facility as unexampled as it is pleasing, we can at once obtain a store of information, which would otherwise require hours of research over the field of an extensive library. Nor is it confined in its character to that of a Dental Dictionary merely. The two sciences are so inseparably blended, that the author has not found his work completed, until he has, at the same time, produced one of the best *Medical* Dictionaries of the day.

It has already become so indispensable a part of our library, that it is a matter past all explanation with us, how we ever got along without it!

We congratulate Dr. Harris that he has been a *pioneer* in this new field of labor. He has produced the first Dictionary of the kind in the world; upon this work alone, the immutability of his name is safely founded, for through all of the changes of time, his name will be associated with that of the art. Dr. H., more than any other writer upon the Dental Art, has placed the profession in a position from which they must ever regard him with gratitude. We consider the Dictionary of Dental Science as his *crowning effort.* Removing as it does, one of the great obstacles to its advancement, it will give a new impulse to the Dental art, which will never cease to be felt while it passes on towards that high degree of perfection which seems to await it.

We commend it alike to the student and practitioner; it will prove invaluable to both.

